# Assessment of the Use of Common Juniper (*Juniperus communis* L.) Foliage following the Cascade Principle

**DOI:** 10.3390/molecules28104008

**Published:** 2023-05-10

**Authors:** Irene Mediavilla, Raquel Bados, Lillian Barros, Virginie Xavier, Tiane C. Finimundy, Tania C. S. P. Pires, Sandrina A. Heleno, Ricardo C. Calhelha, Joana S. Amaral, Andrea Maria Rizzo, David Casini, Giacomo Lombardi, David Chiaramonti, Miguel Cámara, Ana Suárez, Tomás Ardid, Luis Saúl Esteban

**Affiliations:** 1 Centre for the Development of Renewable Energies—Centre for Energy, Environmental and Technological Research, CEDER-CIEMAT, Autovía de Navarra A-15, Salida 56, 42290 Lubia, Spain; raquel.bados@ciemat.es (R.B.); luis.esteban@ciemat.es (L.S.E.); 2 Centro de Investigação de Montanha (CIMO), Instituto Politécnico de Bragança, Campus de Santa Apolónia, 5300-253 Bragança, Portugal; lillian@ipb.pt (L.B.); virginie.xavier@ipb.pt (V.X.); tiane@ipb.pt (T.C.F.); tania.pires@ipb.pt (T.C.S.P.P.); sheleno@ipb.pt (S.A.H.); calhelha@ipb.pt (R.C.C.); jamaral@ipb.pt (J.S.A.); 3 Laboratório Associado para a Sustentabilidade e Tecnologia em Regiões de Montanha (SusTEC), Instituto Politécnico de Bragança, Campus de Santa Apolónia, 5300-253 Bragança, Portugal; 4 Renewable Energy Consortium for Research and Demonstration, RE-CORD, Viale J. F. Kennedy, 182, 50038 Scarperia e San Piero, Italy; andreamaria.rizzo@re-cord.org (A.M.R.); david.casini@re-cord.org (D.C.); giacomo.lombardi@re-cord.org (G.L.); david.chiaramonti@polito.it (D.C.); 5 TOLSA, Calle Núñez de Balboa, 51, 28001 Madrid, Spain; mcamara@tolsa.com (M.C.); asuarez@tolsa.com (A.S.); tardid@tolsa.com (T.A.)

**Keywords:** absorbent, bioactivity, biochar, essential oil, hydrolate

## Abstract

*Juniperus communis* L. is a species commonly grown in regions of the Northern Hemisphere, and is a good candidate to be cultivated in marginal lands. Plants coming from a pruning performed in a natural population located in Spain were used to assess the yield and quality of different products obtained following the cascade principle. A total of 1050 kg of foliage biomass were crushed, steam-distilled, and separated into fractions to produce biochar and absorbents for the pet industry using pilot plants. The obtained products were analysed. The essential oil, with a yield of 0.45% dry basis and a qualitative chemical composition similar to that described for the berries in international standards or monographs, showed antioxidant activity with promising CAA results (inhibition of 89% of the cell’s oxidation). However, regarding antibacterial and antifungal activities, it only inhibited the growth of microorganisms at the maximum concentration tested, 2.5%. Concerning the hydrolate, it did not show bioactivity. Regarding the biochar, whose yield was 28.79% dry basis, interesting results were obtained for its characterisation as a possible soil improver for agronomic purposes (PFC 3(A)). Finally, promising results were obtained regarding the use of common juniper as absorbent, taking into account the physical characterisation and odour control capacity.

## 1. Introduction

A sustainable and circular European bioeconomy is needed to deal with global challenges such as climate change, land and ecosystem degradation, and a growing population. In this sense, modernisation and strengthening of the EU industrial base through the creation of new value chains and greener, more cost-effective industrial processes are of particular importance [1]. However, the production of new biobased products from vegetal biomass entails the use of land, which is a finite and scarce resource, and competition with food production or other necessities such as preservation of habitats, regeneration of ecosystems, or sequestration of carbon, can happen [2]. Then, the production of biomass on abandoned, unused, or severely degraded land (also referred to as marginal land) [3] could be a way to overcome a wide range of land-use challenges [2].

The main objective of the BeonNAT project is to create added-value biobased products within the concept of biorefinery, growing underused tree and shrub species in marginal lands. The BeonNAT biorefinery encompasses refining lignocellulosic biomass into intermediate outputs to be processed into a spectrum of biobased marketable products (vegetal extracts, essential oils, bioplastics, biochar, active carbon, absorbents for pets, particleboard, and pulp for paper), and bioenergy. Prior to the use of cultivated species, different tests are carried out with wild biomass in order to study the different processes and the quality and application of the products obtained. One of the species selected within the project is *Juniperus communis* L. It is an evergreen, perennial, long-lived coniferous plant which has the largest range of any woody plant in the cool temperate geographical regions of the Northern Hemisphere, from the Arctic south, in mountains, to around a latitude of 30° north in Europe, Asia, and North America [4]. It grows in poor soils and harsh environments, being drought- and cold-tolerant, although it requires a minimum of a year’s water precipitation. In its southern distribution area, it can tolerate shade and can be found in the understorey of many forests. Moreover, common juniper can grow on acidic sandy or calcareous soils and favours free-draining soils and rocky outcrops.

Nowadays, the berry cones of *J. communis* and their essential oils are recognised by the European Pharmacopoeia. Berry cones are traditionally well known by folk medicine [5] and different studies on their essential oil have shown its strong antibacterial, antifungal, antiviral, antioxidant, and anti-inflammatory properties [4,6,7]. Some differences have been found between the essential oil of *J. communis* berry cones and leaves with regard to essential oil yield and composition [4,8,9], and most of the studies related to the bioactivity of *J. communis* essential oils have been focused on the oil coming from the berry cones. However, some studies have shown antioxidant and insecticidal properties for the essential oil obtained from the leaves and foliage of this species [10,11,12], but more research work is needed to study the bioactivity of this essential oil in order to know more information about its bactericidal and antifungal activities and complementary studies considering its antioxidant capacity.

After the steam distillation process, different byproducts are obtained, such as distilled vegetal biomass, distillation wastewater, and hydrolate. The distilled vegetal biomass is used as fuel to produce process heat or for composting in some industrial installations. However, other uses, such as extracts [13,14,15] or bio-oil [16] production, are being investigated with different species. To the authors’ knowledge, alternative uses of distilled *J. communis* have not been studied, and the production of new green products, such as biochar and absorbent pellets for the pet industry, is proposed in this work.

Although juniper pyrolysis has been traditionally used to produce tar for different purposes such as gluing materials, waterproofing decoration, medicine, and cosmetics [17], no investigation has been found concerning the production of biochar, which is an interesting product from the point of view of soil improver for agronomic purposes and as strategic solution for carbon capture and storage.

Regarding the absorbents for the pet industry, the cat litter market is nowadays led by minerals, more specifically by clays—such as bentonite, sepiolite, and attapulgite—due to their high absorption and odour control capacity. However, a new biodegradable, eco-friendly mindset is surfacing in consumers and pushing cat litter manufacturers to enlarge their products on offer. The most known and sold product in this matter is wood pellets, which are made of sawdust obtained from heat-treated wood waste to remove toxins, oils, and allergens. Although wood pellets are well known for their high absorption capacity, they disintegrate in contact with liquid and, in principle, do not present a specific structure (as mineral do) to retain bad-odour-causing molecules and, consequently, ammoniacal odours can be detected [18]. Even so, other types of vegetal/plant-based fibres, such as those obtained after *J. communis* distillation, may be more efficient in odour control and capable of retaining or neutralising bad odour molecules.

On the other hand, hydrolate is a product of condensation after the essential oil isolation procedure. It is a colloidal suspension composed of a continuous phase, the distilled water, and a dispersed phase, which is an emulsion of essential oil droplets (usually below 1 g/L) and water-soluble components, namely, oxygen-containing compounds [19,20]. Hydrolates are easy to obtain, being formed in high amounts in the essential oil industry and, considering sustainability, there is a growing interest in giving added value to this byproduct. They have been historically used in traditional medicine in Mediterranean countries, as refreshing drinks and flavourings [21], and in cosmetics [22]. Additionally, investigations focused on their composition and biological properties have recently demonstrated their antimicrobial [21] and antioxidant [22] activities. Regarding the *J. communis* hydrolate, very few authors have published studies on its bioactivity, such as Oral et al. [23] who studied its antibacterial capacity. Considering the high amounts of hydrolate obtained during the steam distillation process, the study of possible uses of that hydrolate is important to complement the studies carried out on the essential oil.

Finally, the distillation wastewater results from condensation of some of the steam passing through the biomass, and it is collected at the bottom of the container. Currently, it is not a usable product and is released as a waste product. Very few studies have been carried out on its possible applications [19,24].

The objective of this work is to assess the yield and quality of different products obtained from *Juniperus communis*, such as essential oil, hydrolate, biochar, and absorbents, following the cascade principle.

## 2. Results and Discussion

### 2.1. Conditioning of Biomass and Steam Distillation

All the biomass collected was shredded at 20 mm and a sample was separated in order to analyse its moisture content, which was 42.5%.

Two samples of 400 kg of shredded material were distilled in the pilot plant described in Section 3.3 and the average essential oil yield was 0.45% (standard deviation: 0.035), expressed in weight percentage of dry essential oil referred to dry juniper. This value was of the same order of magnitude as those obtained in a previous work with *J. communis* collected in May 2018 and June 2020 in an area next to the area considered in this work and distilled in a plant with a capacity of 30 L [11]. However, the yields reported for *J. communis* foliage distillation in other studies [25,26,27,28,29] are variable (0.05–2.43%), although these results were obtained on a laboratory scale using a Clevenger apparatus with steam distillation or hydrodistillation and are not comparable to the tests considered in the present work.

Concerning the hydrolate and distillation wastewater production, the byproduct yields were 35.9% (standard deviation: 4.9) for hydrolate and 35.8% (standard deviation: 7.2) for wastewater, expressed in weight percentage of byproduct referred to dry juniper biomass.

**Figure 1:**
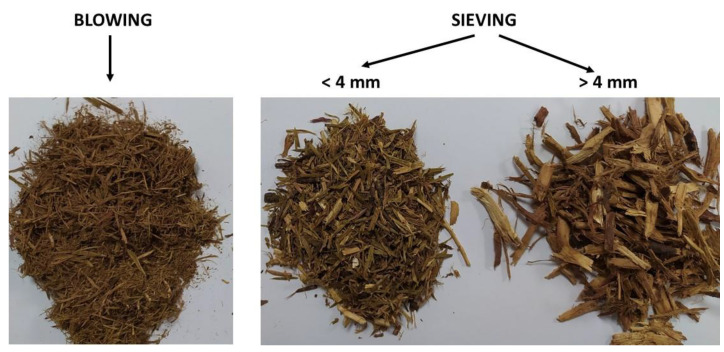
Biomass fractions obtained after the separation process.

After the distillation, the distilled biomass was air-dried, reaching a moisture content of 10.3%, and it was separated into three fractions using a sieving (4 mm) and blowing step. Two tests using 200 kg of air-dried distilled biomass each were performed, obtaining the following results, expressed as average value in weight percentage (wet basis): 11.9% (standard deviation: 0.42) of biomass from blowing, 54.6% (standard deviation: 1.04) of biomass < 4 mm from sieving, and 33.5% (standard deviation: 1.46) of biomass > 4 mm from sieving. Figure 1 shows some pictures of the biomass fractions obtained.

**Figure 2:**
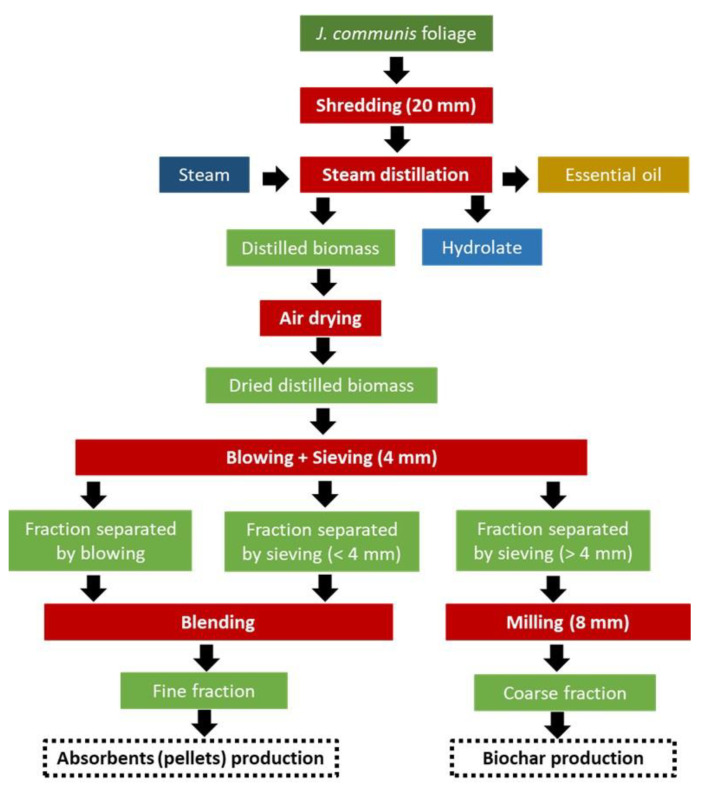
Block diagram of the biomass conditioning process and cascading.

After the separation process, the fine fraction, composed of the biomass from blowing and the biomass with size below 4 mm obtained after sieving, corresponded to 66.5%, and the coarse fraction, composed of the biomass with size above 4 mm obtained after sieving, was 33.5% of the total biomass distilled. Taking into account the cascade defined in this work (Figure 2), pelletisation of the fine fraction was carried out, whereas milling at 8 mm of the coarse fraction was performed prior to pyrolysation.

### 2.2. Essential Oil and Hydrolate Composition

The essential oils obtained in the two steam distillation tests were blended and analysed using GC–MS. All the components identified and their quantification using the relative area percentage can be found in Mediavilla et al. [30], while Table 1 shows only the main compounds detected (>5%) in addition to those included in the European Pharmacopoeia for *Juniperus communis* berries essential oil and the standard ISO 8897:2010.

The characterisation of the analysed essential oil showed that monoterpene hydrocarbons were the most abundant compounds (68.1%), followed by sesquiterpene hydrocarbons (21.5%), oxygenated monoterpenes (5.7%), and, finally, oxygenated sesquiterpenes (0.8%). The main compounds (>5%) were four monoterpene hydrocarbons, namely, α-pinene (18%), limonene (16%), sabinene (11%), and myrcene (5.9%), and one sesquiterpene hydrocarbon, i.e., cis-thujopsene (5.1%). Comparing the obtained composition with the limits considered by the European Pharmacopoeia and the standard ISO 8897:2010, both referred to *J. communis* berries, it can be observed that the composition of the foliage essential oil shown in this work was close to meeting the limits. However, it can be noted that α-pinene was slightly below the limit (20.0% in the European Pharmacopoeia and 25.0% in the ISO standard), limonene was higher than the limit (12.0% in the European Pharmacopoeia and 8.0% in the ISO standard), and α-phellandrene (2.2%) was higher than the limit considered by the European Pharmacopoeia (1.0%). Comparing this characterisation with that obtained in a previous study of steam distillation of *J. communis* foliage collected in Spain, it can be observed that the distribution of percentages among the different component groups was similar, although with higher contents of sabinene and lower contents of limonene and myrcene [11]. Taking into account that the composition of *J. communis* essential oil changes throughout the year [27,32], being also affected by the proportion of leaves and berries contained in the sample distilled [8,9,10], more studies are required. They should comprise different locations and collecting periods to establish if the essential oil obtained from steam distillation of the foliage could fulfil the limits set out for the berries essential oil as described in the European Pharmacopoeia and/or the standard ISO 8897:2010.

The hydrolates obtained in the two steam distillation tests were blended and extracted, obtaining a yield of 0.18% (*w*/*w*). Afterwards, the extracted fraction was analysed using GC–MS. The components identified and their quantification using the relative area percentage are shown in Table 2.

The analysed hydrolate showed a qualitative and quantitative profile different from that of the essential oil since it contained mainly oxygenated monoterpenes. In the hydrolate, terpinen-4-ol was the most abundant compound (58%), followed by α-terpineol (10%). All the remaining identified compounds represented less than 5% of the total composition. Despite appearing in low amounts, some of the compounds were identified only in the hydrolate, probably due to their higher solubility in the aqueous phase since those were mainly small-weight alcohols and ketones.

### 2.3. Bioactivity of Essential Oil and Hydrolate

With regard to the antioxidant activity, the essential oil presented an EC50 of 1.14 ± 0.09 mg/mL in the reducing power assay (positive control: Trolox = 41 µg/mL) and inhibited 89% of the cells’ oxidation in the CAA assay. Similar to the hydrolate, the essential oil also presented hepatotoxicity at a concentration of 179 ± 9 µg/mL. In all the bioactive assays performed, the hydrolate did not present activity at the higher concentration tested (2000 µg/mL), neither antioxidant nor antimicrobial activity, which is possibly explained by the high dilution factor of these products. Still, the hydrolate presented some hepatotoxicity (219 ± 13 µg/mL).

The antibacterial activity of the essential oil was tested in a concentration range from 2.5% (*v*/*v*) to 0.039% (*v*/*v*) and the clinical bacteria were not sensitive at the highest concentration tested (Table 3). Better results were obtained against the foodborne bacteria (Table 4) since the Gram-negative *S. enterica* and the Gram-positive *B. cereus* were inhibited by the *J. communis* essential oil at the highest concentration tested 2.5% (*v*/*v*). The essential oil at 2.5% (*v*/*v*) was also able to inhibit the growth of the two tested fungi (Table 5).

According to Borges et al. [33], the concentration of α-pinene is directly related with antibacterial properties. Comparing the results obtained by Xavier et al. [34], the oil of the same species obtained in the same region showed α-pinene as the major compound, in concentrations about twice as high, and consequently results with greater antibacterial potential. Gonçalves et al. [35] found that the essential oil derived from *J. communis* biomass displayed potent inhibitory activity against *E. coli*, with concentrations of 1.25–2.5 mg/mL. However, no notable inhibitory activities were observed against other Gram-negative bacteria, including *P. mirabilis, K. pneumoniae*, *P. aeruginosa*, and *M. morganii*. There was a slight activity against *L. monocytogenes* and methicillin-resistant *S. aureus*. Dumitrescu et al. [36] discovered that commercially available essential oils of *J. communis* were active against *E. coli* and *S. aureus*, but not *P. aeruginosa*. The MIC values for various *Aspergillus* species ranged from 11 to 14 mg/mL. Meanwhile, the essential oil from *J. communis* berries, as per Cavaleiro et al. [37], showed an MIC of 10 µL/mL against *A. fumigatus*. As for antioxidant activity, Gonçalves et al. [35] stated that the essential oils tested exhibited the ability to scavenge ferric species (0.47 and 1.11 mM FeSO_4_·7H_2_O). When compared to Xavier et al. [34], the oil from the same species in the same region demonstrated an EC_50_ of 1.35 ± 0.20 mg/mL with reducing power assay and 68.79 ± 3.34% oxidation inhibition with CAA assay. The essential oil also showed hepatoxicity (212.03 ± 23.26 ug/mL). Jakubczyk et al. [22] found that juniper hydrolate contained 2 292.98 µM Fe(II)/L, while other plants’ hydrolates had higher antioxidant potential depending on the species, origin, part of the plant, and preservation method used. The juniper essential oil and hydrolate may possess bioactive properties depending on various factors. However, caution must be exercised while using them due to their potential toxicity towards liver human cell lines.

### 2.4. Pyrolysis and Biochar Characterisation

The coarse fraction obtained after the separation of distilled juniper biomass (Figure 2) was pyrolysed in the pilot scale plant described in Section 3.6, and a biochar yield of 28.79% by dry weight was obtained. Biochar mass yield (the solid fraction resulted by the thermochemical conversion of biomass through the pyrolysis process) depends strictly on the feedstock characteristics, the plant used, and the process parameters chosen (heating rates, maximum temperature, and residence times) [38,39]. SPYRO pilot plant, through the slow pyrolysis process, maximises the mass yield of high-quality char (commonly about 30% by dry weight), while minimising the production of liquid and gaseous products.

The biochar produced showed a total carbon content of 87.8% *w*/*w* d.b. and a fixed carbon content of 78.4% *w*/*w* d.b. As reported by Leng et al. [40], biochar fixed carbon is closely related to stable C content. Moisture content was 1.8% *w*/*w* w.b., and volatile matter and ash content resulted, respectively, in 13.0 and 8.7% *w*/*w* d.b. Fixed carbon content and H/C molar ratio indicate a good degree of carbonisation and stability for the biochar obtained [40,41]. H/C molar ratio values fluctuate depending on the feedstock and the process used; generally, materials with low H/C molar ratio represent products with higher stability and resistance to degradation, similar to in this case, where the molar H/C ratio resulted in 0.35.

**Figure A1:**
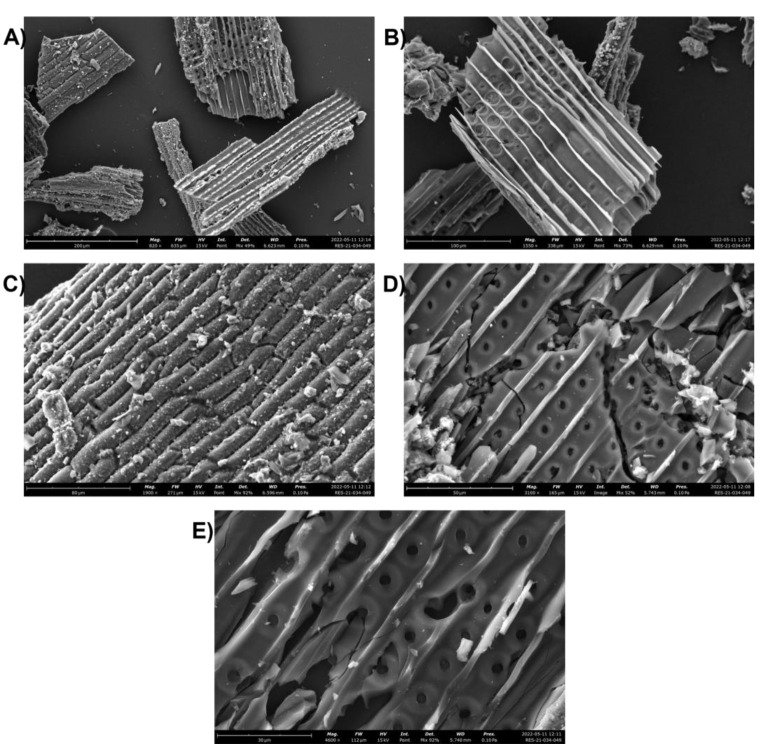
*J. communis* biochar analysed through SEM. (**A**) 200 µm view. (**B**) 100 µm view. (**C**) 80 µm view. (**D**) 50 µm view. (**E**) 30 µm view.

The complete set of proximate and ultimate analysis, elements, and organic pollutants content can be found in Appendix A (respectively, in Table A1, Table A2, and Table A3), as well as the SEM pictures (Figure A1). Biochar porosity analysis can be found in Table A4 in Appendix A, both using BET methodology and mercury porosimetry for the specific surface area and pore volume evaluation. In particular, results with the mercury porosimeter show a volume porosity of 66% and a specific volume intrusion of 1.59 cm^3^/g in the range of meso- and macroporosity.

The biochar water-holding capacity test at 0.01 bar resulted in 141.6% *w*/*w* wet basis. According to Usevičiūtė and Baltrėnaitė-Gedienė [42], wood-based biochars have a wide range of sorption capacity values, mainly related to the feedstock characteristics and to the conversion process, commonly comprising about 1 to about 3 mL/g; the biochar obtained from *J. communis* showed a water-holding capacity of 1.41 mL/g.

The high carbon content, resistant to thermochemical and biological degradation, and only marginally subject to mineralisation by microorganisms [40,43], coupled with the water-holding ability that resulted, represent interesting results for the application of the product obtained as an effective soil improver for agronomic purposes and, at the same time, a potential innovative carbon capture and storage strategic solution (as indicated in the IPCC report released in 2019 [44]. The modifications to Annexes II, III, and IV of the EU Reg. 2019/1009 for fertilising products included the possibility to classify pyrolysis solid residue (biochar) as Component Material Category n.14 (CMC 14). In the Table 6 and Table 7 are presented the compliance of the biochar produced with CMC 14 threshold and the Product Function Category n.3A (PFC 3A) that permits characterisation of the product as a soil improver.

### 2.5. Pelletisation and Absorbent Pellets Characterisation

The fine fraction obtained after the separation of distilled juniper biomass (Figure 2) was pelletised with a moisture content of 12.8%. Concerning the pelletisation process, a specific mass flow of 7.6 kg of dry matter per hour and per kW of drive power and a specific energy of 78 kWh per t of dry matter were obtained. When comparing these values with previous works carried out in a pilot plant to produce solid biofuels, the specific mass flow was higher (27%) than the values obtained with a shrub biomass (*Genista cinerascens* L.) [45] and pine sawdust [46], and the specific energy was lower (34%). These values show that the yield of pelletisation of the fine fraction of *J. communis* separated following the process described in Section 3.2 is higher than the value corresponding to a biomass typically used to produce solid biofuels, such as pine sawdust, or a shrub biomass, such as *G. cinerascens*. This fact could be due to the higher bark content of the fine fraction obtained after the separation process (see Figure 1), since some authors have demonstrated that a higher bark content increases pellet production [47,48].

Regarding the use of these pellets as cat litter, the analysis results can be found in Table 8.

**Figure 3:**
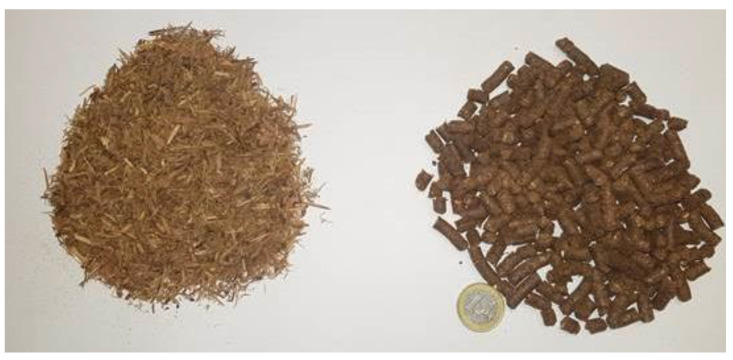
Appearance of *J. communis* material before and after the pelletisation process.

In terms of appearance (Figure 3), the analysed *J. communis* pellets showed well-defined cylindrical shape and darker colour (reddish dark brown with cream–yellowish spots) than that of the wood pellets mostly found in the market. Concerning the results of dry particle size distribution, the pellets were sized between the limits considered as reference. Moreover, it must be noted that the particle size distribution was homogeneous (5.6–6.3 mm), and this fact makes the product more attractive for consumers.

Moisture content, bulk density, and water absorption capacity showed values included in the limits considered as reference. Therefore, the *J. communis* pellets are stable from the point of view of their moisture content, lightweight, and highly absorbent, characteristics demanded in the market for pet absorbents.

Regarding the dust content, although traditional litters of mineral origin can reach values up to 50 u.a., commercial plant-based cat litter is not usually dusty, with values around 8–14 a.u. Consequently, the result obtained with *J. communis* pellets (7 a.u.) was satisfactory.

Durability evaluates the resistance of pellets to repeated beating (transport and handling) and, ideally, in the case of cat litter pellets, it should be higher than 95.0%. However, the *J. communis* pellets analysed showed a durability of 88.9% and, consequently, improving this parameter would be worthy. It must be noted that mechanical durability not only depends on the biomass used but also on the die compression used during the pelletisation process [46]. Then, although this parameter does not fulfil the limits considered as reference, it could be improved through setting the pelletisation process parameters.

Finally, the odour control result, expressed as the NH_3_ concentration that is not retained/neutralised by the material and measured 4 h after the addition of aged real cat urine, was 0 ppm, compared with 16 ppm, which was measured with commercial wood pellets. Consequently, the *J. communis* pellets analysed have very high deodorising capacity.

## 3. Materials and Methods

### 3.1. Vegetal Material

**Figure 4:**
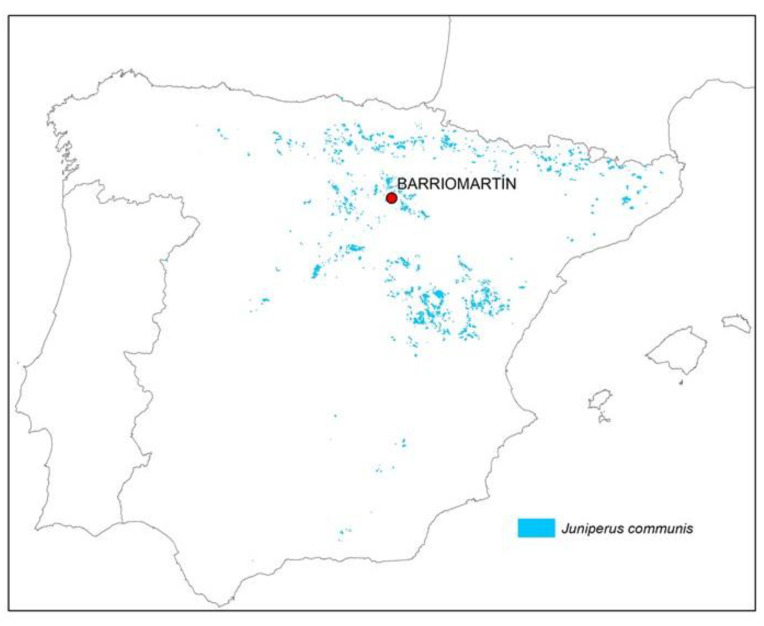
Distribution of natural populations of *J. communis* in Spain (based on MFE25 [49] and MFE200 [50]).

Natural populations of *Juniperus communis* in Spain are shown in Figure 4. One of these populations, located in a mountain area in Spain (Barriomartín; UTM coordinates: 30T 545081; 4649553), was selected. This location has an altitude of 1407 m (a.s.l.) and its vegetation is mainly composed of forest and heath. Regarding the marginality of the site, predominantly poor substrate and low rooting depth can be highlighted.

A sample of 1050 kg of foliage (50% of male plants and 50% of female plants) coming from plants with stem diameter below 50 mm was collected on 6 and 7 April 2021. The objective of using plants with this stem diameter was to reproduce the pruning process in a plantation of this species, since common juniper has good response to trimming.

### 3.2. Conditioning of Biomass

The collected foliage biomass was crushed (20 mm) using a shredder (90 kW, slow-rotating single-shaft type). The freshly ground biomass was distilled in a steam distillation pilot plant and the distilled biomass was air-dried at temperature below 40 °C and was separated into two fractions by means of a sieving and blowing step (0.37 kW, gyratory-reciprocating motion screener). The sieve used had a mesh of 4 mm, and a fine fraction composed of the particles with size below 4 mm and blown particles and a coarse fraction composed of the particles with a size above 4 mm were obtained. Finally, the coarse fraction was milled in a hammer mill (11 kW) at 8 mm. Subsamples were taken along the process to determine the moisture content in an oven at 105 °C until they reached a constant weight, following the standard ISO 18134-2:2017. Figure 2 shows the different steps followed.

### 3.3. Steam Distillation

A steam distillation pilot plant described in a previous work [30] was used to distil the *J. communis* biomass. Two batch distillations of 400 kg each were performed with a duration of 6 h (time was measured from the moment the first drop of distillate fell). The duration of the tests was defined considering that the quantity of essential oil obtained in the last 1.5 h was below 5% of the total essential oil obtained during the whole process. The essential oil samples were weighed and dried using anhydrous sodium sulphate and, after filtration, stored at 4 °C until further analysis. The oil yield for each sample was calculated as a percentage (*w*/*w*) on a biomass dry weight basis.

The hydrolate and the residual water obtained during the distillation process were weighed. A sample of hydrolate was separated and stored at 4 °C until further analysis. The hydrolate and residual water yields were calculated as a percentage (*w*/*w*) on a biomass dry weight basis.

### 3.4. Essential Oil and Hydrolate Characterisation: Chemical Composition

One sample of essential oil was obtained, blending the two samples of essential oil produced in the steam distillation tests, and it was analysed by gas chromatography–mass spectrometry (GC–MS) on a Shimadzu GC-2010 Plus chromatograph equipped with AOC-20iPlus automatic injector (Shimadzu, Canby, OR, USA) and an SH-RXi fused-silica column (30 m × 0.25 mm i.d., film thickness 0.25 μm; Shimadzu, Canby, OR, USA) according to the conditions described by Spréa et al. [51].

One sample of hydrolate was obtained, blending the two samples of hydrolate produced in the steam distillation tests, and it was analysed by GC–MS under the same conditions. The hydrolate (15 mL) was extracted using liquid–liquid extraction with organic solvents. Two cycles of diethyl ether (15 mL) and 3 cycles of hexane (15 mL) were performed. Anhydrous sodium sulphate was added to guarantee that no water from the hydrolate remained in the organic fraction. The extracted fraction was filtered and evaporated until dry. The remaining fraction was diluted in 2 mL of hexane (HPLC grade), filtered with syringe filter, and stored in the freezer until further analysis.

### 3.5. Essential Oil and Hydrolate Characterisation: Bioactive Evaluation

Both essential oil and hydrolate were evaluated for their antimicrobial activity, antioxidant activity, and hepatotoxicity.

#### 3.5.1. Antimicrobial Activity

The antibacterial activity of *J. communis* essential oil and hydrolate was evaluated against clinical isolates, foodborne bacteria, and fungal species. The clinical isolates were obtained from patients hospitalized in various departments at the northeastern local health unit (Bragança, Portugal) and Hospital Center of Trás-os-Montes and Alto Douro (Vila Real, Portugal). Five Gram-negative bacteria, *Escherichia coli* (isolated from urine), *Proteus mirabilis* (isolated from wound exudate), *Klebsiella pneumoniae* (isolated from urine), *Pseudomonas aeruginosa* (isolated from expectoration), and *Morganella morganii* (isolated from urine), and three Gram-positive bacteria, *Enterococcus faecalis* (isolated from urine), *Listeria monocytogenes* (isolated from cerebrospinal fluid), and methicillin-resistant *Staphylococcus aureus* (MRSA) (isolated from expectoration), were tested. Concerning the food contaminants, the essential oil and hydrolate were tested against five Gram-negative bacteria, namely, *Enterobacter cloacae* (ATCC 49741), *Escherichia coli* (ATCC 25922), *Pseudomonas aeruginosa* (ATCC 9027), *Salmonella enterica* subsp. (ATCC 13076), and *Yersinia enterocolitica* (ATCC 8610), and three Gram-positive bacteria, namely, *Bacillus cereus* (ATCC 11778), *Listeria monocytogenes* (ATCC 19111), and *Staphylococcus aureus* (ATCC 25923).

Additionally, the essential oil and hydrolate were also tested against two fungi: *Aspergillus brasiliensis* and *Aspergillus f*umigatus. *A. brasiliensis* is a common fungal species found in various environments, including soil, food, and indoor settings, such as industrial plants. Although it is a frequent food contaminant, it rarely causes human illness. In contrast, *A. fumigatus* can colonize the upper respiratory tract of individuals with asthma, causing severe symptoms, reduced lung function, and occasionally resulting in severe diseases.

Food strains and fungal species were purchased at Frilabo, Porto, Portugal.

The antimicrobial activity was evaluated through the broth microdilution method, based on the methodology described by Falcão et al. [6] and later adapted to a microdilution method by Xavier et al. [34].

#### 3.5.2. Antioxidant Activity

The antioxidant capability of the essential oil and hydrolate was determined using the reducing power (RP) [52] and the cellular antioxidant activity (CAA) [53] assays. All human or animal cell lines used in this manuscript were commercially available and were purchased from different authorized cell line resources, including the German Collection of Microorganisms and Cell Cultures (DSMZ) and the European Collection of Authenticated Cell Cultures (ECCAC). In order to maintain high scientific standards, all procedures were performed according to the best practices observed in the Guidance on Good Cell Culture Practice (GCCP). The efficacy of the antioxidant treatments was subsequently measured by the percentage reduction in fluorescence for CAA measurement at an excitation wavelength of 485 nm.

#### 3.5.3. Hepatotoxicity

The hepatotoxicity of the essential oil was performed following a sulforhodamine B (SRB) assay previously described by the authors [54].

### 3.6. Pyrolysis and Biochar Characterisation

The coarse fraction obtained after the conditioning of the distilled biomass (Figure 2) was pyrolysed using an innovative pilot plant composed of a continuous auger-type pyrolysis reactor (SPYRO) and by an in-series condensation unit. SPYRO is designed to process up to 3 kg/h of biomass and can be operated up to 600 °C. The rotating speed of the reactor screw can be easily adjusted to vary the solids residence time, from few minutes up to 1 h. A total of 4220 g of wet feedstock were processed with an average heating rate of 17.8 °C/min and a residence time of about 30 min at the maximum pyrolysis process temperature of 550 °C (slow pyrolysis process). The charcoal was collected by gravity in a sealed vessel, while volatiles were separated into a pyrolysis liquid that was collected in glass bottles, and an off-gas stream composed of permanent gas.

The biochar obtained was characterised in terms of proximate and ultimate analysis, elements content, organic pollutants, conductivity, specific surface area, pH, and water-holding capacity at 0.01 bar (results are presented in Appendix A). Biochar sample was also analysed using a scanning electron microscopy.

The moisture, ash, and volatile matter contents were analysed using a Leco Thermogravimetric Analyser TGA 701. The moisture content was measured at 105 °C following the UNI EN ISO 18134-2 standard, the ash content was measured at 550 °C according to UNI EN ISO 18122, and the volatile matter at 900 °C according to UNI EN ISO 18123. The fixed carbon was calculated according to UNI EN 1860-2. The total carbon (C), hydrogen (H), and nitrogen (N) contents were analysed in a Leco TruSpec CHN analyser according to UNI EN ISO 16948.

The sulphur and chlorine contents were analysed by ion chromatography according to UNI EN ISO 16994 in a Metrohm 883 Basic IC Plus.

The inorganic carbon content was measured according to the current Italian decree n.7276, addition n.13 of the 31 May 2006 (Dietrich–Fruhling calcimeter); and the organic carbon was calculated as the difference between the total (CHN) and the inorganic carbon.

The water-holding capacity of the biochar was determined according to Italian D.M. 1 August 1997 Method 5, which refers to soil physical analysis, and adjusted to be suitable for biochar analysis. 

Microelement and macroelement contents were analysed through an ICP (Agilent 4200 MP-AES, Santa Clara, CA, USA), previously calibrated with a multielemental standard solution. For the former mineralisation step, 0.05 g of biochar were weighed and 3 mL of H_2_O_2_ and 8 mL of HNO_3_ were added, then the samples were mineralised in a Milestone Start D Microwave Digestor System. At the end of the step, HNO_3_ at 1% concentration was added to the collected mineralised samples, reaching 20 mL in volume, and then the solutions were analysed in the ICP.

The concentration of toxic metals, such as Cd, As, Hg, and Tl were analysed according to EN 13657 + EN ISO 7294, while Cr VI was analysed according to EPA 3060 A + EPA 7196. Other harmful compounds (PAH, polychlorinated biphenyls, dibenzodioxins and dibenzofurans) were analysed, respectively, through EPA 3540 C + EPA 3630 C + EPA 8270 E for PAHs, EPA 1668 C for polychlorinated biphenyls, and EPA 1613 B for polychlorinated dibenzodioxins and dibenzofurans.

The biochar specific surface area was determined according Brunauer–Emmett–Teller method (BET), by N_2_ adsorption isotherms, in a Quantachrome NOVA 2200E instrument. All measurements were performed on 60 mg of samples preliminarily dried at 200 °C for 48 h and then degassed under vacuum (200 °C for 24 h). Meso- and macroporosity, in terms of specific surface area and pore volume, were investigated using a Micromeritics AutoPore V 9600 mercury porosimeter.

The pH was measured according to UNI EN ISO 10390 in 0.1 M of CaCl_2_ (1:5 *v*/*v*) through a Metrohm 827 pH meter.

Electrical conductivity was determined according to ISO 11265 using a Thermo Scientific (Waltham, MA, USA) COND6+ conductivity meter after water extraction and filtration.

Finally, the SEM experiment was conducted using a Thermo Scientific Phenom XL G2. Measurements were conducted on uncoated samples.

### 3.7. Pelletisation and Absorbent Pellets Characterisation

Finally, the fine fraction obtained after the biomass conditioning (Figure 2) was used to produce pellets in a small pilot plant. The pellet press, KAHL 14-175, is a flat-die machine with dosing hopper and variable speed. With an engine of 3 kW, this press can produce 20–40 kg pellets/h. The flat die used has a diameter of 175 mm, holes with 6 mm of diameter, and a die compression (length of the inlet cone + straight channel of the hole) of 28 mm. Two rollers with a width of 15 mm and rotating speed between 0.5 and 0.8 m/s force the material to move through the flat die.

With the objective of producing high-quality pellets and optimising the operation of the pellet press with stable power demand and low vibration, the flows of water and biomass fed to the machine were modified. Once these conditions were achieved, the steady state was reached and it was maintained for 40 min. Once the pellets were cooled down, they were manually sieved with a 3.15 mm sieve in order to simulate an industrial process removing the fines content.

Two process variables were recorded in the pelletisation tests: the specific mass flow, which is expressed in kg of dry matter per hour and per kW of drive power, and the specific energy, which is expressed in kWh per t of dry matter. In the calculation of the specific mass flow, the mass of pelletised material, in kg of dry matter, is divided by the time used to pelletise it, expressed in hours, and by the power of the pellet press, which is 3 kW. On the other hand, the specific energy is calculated, considering the active electric energy required to pelletise the material, divided by the mass of pellets obtained, expressed in t of dry matter.

Since the final use of the obtained pellets is as absorbent cat litter, the following characteristics were determined:

- Appearance. This is visually determined by observing and writing down all the particularities referring to the nature of the sample: colour, particle size distribution, structure, and porosity of the particles, as well as dust generation, possible blend of raw materials, and possible additives presence.
- Dry particle size distribution (method based on UNE 22-162 and ISO 2591-1). This is the relative percentage of different size particles conforming a product. This trial is performed by dry-sifting throughout a series of standardised screens, and calculation of the percentage of material that remains on each screen.
- Moisture content (own method). This provides information on the quantity of water naturally contained in the sample, and this test is carried out by means of a halogen moisture analyser (Mettler Toledo, HG53, Columbus, OH, USA) at a temperature of 145 °C.
- Dust content (own method). This parameter measures the dust cloud generated by the cat litter when pouring the material in terms of the cloud’s height and time that it takes to disappear. A sample is placed in a funnel at a known distance from the ground and dropped. From the moment that the sample ends, the time needed for dissipating the cloud of dust generated is measured. Moreover, the density and height reached by the dust cloud are also assessed and the final result is obtained in arbitrary units.
- Bulk density (method based on AFNOR NFT 73-405, UNE 55-516-89, ISO 697 and BS 3762). This is calculated by dividing the mass of pellets confined by a certain volume.
- Water absorption (method based on AFNOR NF V 19-002). This is determined by means of the Westinghouse method, which tests the static water absorption capacity of the vegetal pellets when fully immersed and wet until saturation.
- Durability (own method). This parameter determines the resistance to pellet breakage and fines generation. It is measured by the durability index, which represents the pellet that remains intact after shaking 500 g at 50 rpm for 10 min in a Pfost tumbling box.
- Ammonia absorption capacity (own method). This is calculated using a rapid laboratory method for punctually assessing the deodorising capacity of cat litter against ammoniacal odours from urine. The technique is based on the use of suitably defrosted aged urine, which is mixed with the litter, allowing absorption. After 4 h, ammonia release is measured as NH_3_ gas concentration (ppm), combined with olfactory perception. For measuring ammonia, a Dräger Accuro model gas detector pump combined with control tubes for ammonia determination (type ammonia 5/a) and 500 mL bottles with cap are used. The results are expressed as follows:
  - High deodorant capacity: when no unpleasant odour is observed and the concentration of NH_3_ emitted by the sample is less than 5 ppm.
  - Medium deodorant capacity: when a slight odour of urine is perceived and the concentration of NH_3_ emitted is between 5 and 70 ppm.
  - Low deodorant capacity: when a clear smell of urine is perceived and the concentration of NH_3_ emitted is between 70 and 210 ppm.
  - No deodorant capacity: when the intense smell of urine is annoying and the concentration of NH_3_ emitted by the sample is greater than 210 ppm.

## 4. Conclusions

Common juniper obtained from pruning seems to be an appropriate raw material to produce essential oil, biochar, and absorbents following the cascade principle, which could be an incentive to grow this species or to increase its forest management. Yields of 0.45% and 35.9% on dry weight were obtained for essential oil and hydrolate, respectively, while the distilled biomass was separated into two fractions, dedicated to biochar (33.5%) and absorbents (66.5%) production.

To the best of our knowledge, this work describes for the first time the chemical composition of common juniper foliage hydrolate, evidencing oxygenated monoterpenes as the major group of compounds. The essential oil showed antioxidant activity with promising CAA results. From all the tested bacteria, the essential oil only showed bacteriostatic action against *S. enterica* and *B. cereus* at the maximum concentration tested; furthermore, it was able to inhibit the growth of the two tested fungi. In general, the qualitative chemical composition of the essential oil obtained from juniper foliage was similar to that described for the berries in the European Pharmacopoeia and the standard ISO 8897:2010; however, it did not comply with all criteria described for the berries essential oil since the amounts of limonene, α-pinene, and α-phellandrene were not in the value range described. Considering that the chemical composition can vary with several factors such as climate, soil, and plant sex, among others, more research is needed to determine whether the essential oil extracted from the foliage of *J. communis* might meet the requirements set for the berries essential oil as described in the international standards or monographs.

Thanks to the thermochemical conversion of 33.5% of the distilled biomass (10.3% moisture content) through a slow pyrolysis pilot unit, it was possible to obtain, with a mass yield of 28.79% on dry weight, a quality biochar with a high stable carbon content. The water retention value of 1.42 mL/g resulted as coherent with the total intrusion volume resulted in the range of meso- and macroporosity. This property coupled with the high recalcitrant carbon content of the biochar represent interesting results for the potential application of this product as an effective soil improver for agronomic purposes and, at the same time, a potential innovative strategic solution for carbon capture and storage. The complete characterisation of the sample shows its compliance with EU legislation for fertilising products as a soil improver, PFC 3(A).

Finally, concerning the use of common juniper as absorbent, high yield was obtained during pelletisation (7.6 kg of dry matter per hour and per kW of drive power and 78 kWh per t of dry matter). Moreover, considering the use of these pellets as cat litter, appropriate values regarding moisture content, bulk density, water absorption, dust content, and odour control were obtained, resulting in a very interesting product for this purpose. However, pellets durability should be improved in order to meet the requirements established for this kind of product, and, consequently, further investigation on the pelletisation process is needed.

## Figures and Tables

**Table 1:** Chemical composition of the *J. communis* essential oil.

Compound	Rt (min)	LRI ^a^	LRI ^b^	Relative % ^c^
α-Pinene	14.05	928	932	18 ± 2
Sabinene	16.11	968	969	11 ± 1
β-Pinene	16.24	970	974	1.69 ± 0.02
Myrcene	17.08	986	988	5.9 ± 0.4
α-Phellandrene	17.70	998	1002	2.2 ± 0.1
Limonene	19.00	1023	1025	16 ± 2
Terpinen-4-ol	26.54	1172	1174	3.6 ± 0.1
Bornyl acetate	31.72	1281	1284	0.29 ± 0.05
β-Caryophyllene	37.72	1416	1417	2.2 ± 0.3
cis-Thujopsene	38.22	1428	1429	5.1 ± 0.5
Humulene	39.15	1451	1452	1.5 ± 0.2
Germacrene D	40.28	1478	1480	1.8 ± 0.3

^a^ LRI: linear retention index determined on a DB-5 MS fused silica column relative to a series of n-alkanes (C8–C40). ^b^ LRI: linear retention index reported in the literature [31]. ^c^ Relative % is given as average ± standard deviation.

**Table 2:** Chemical composition of *J. communis* hydrolate (oil fraction extracted).

Compound	Rt (min)	LRI ^a^	LRI ^b^	Relative % ^c^
2-Hexenal	10.14	848	846	0.089 ± 0.002
3-Hexen-1-ol	10.27	851	850	0.89 ± 0.02
2-Hexen-1-ol	10.82	863	859	0.219 ± 0.02
1-Hexanol	10.93	865	863	0.34 ± 0.03
α-Pinene	14.06	929	932	0.73 ± 0.04
Camphene	14.80	943	946	0.20 ± 0.02
1-Octen-3-ol	16.52	976	974	0.27 ± 0.03
β-Myrcene	17.12	988	988	0.14 ± 0.01
α-Terpinene	18.79	1020	1014	0.1281 ± 0.004
p-Cymene	19.01	1024	1020	0.55 ± 0.01
Limonene	19.12	1026	1025	0.44 ± 0.03
γ-Terpinene	21.29	1068	1054	0.28 ± 0.02
trans-Linalool oxide	22.12	1084	1084	0.18 ± 0.01
Linalool	23.78	1117	1095	1.4 ± 0.1
2-p-Menthen-1-ol	24.01	1122	1118	0.165 ± 0.003
α-Campholenal	24.47	1131	1122	0.17 ± 0.01
trans-p-Mentha-2,8-dien-1-ol	24.67	1135	1133	2.8 ± 0.1
trans-Pinocarveol	24.94	1141	1135	3.2 ± 0.1
Sabinaketone	25.75	1157	1154	0.152 ± 0.002
BorneoL	26.05	1163	1165	0.9 ± 0.1
p-Mentha-1,5-dien-8-ol	26.08	1163	1166	0.67 ± 0.03
Terpinen-4-ol	26.69	1176	1174	58 ± 2
Cryptone	26.99	1182	1185	3.7 ± 0.1
α-Terpineol	27.27	1187	1186	10 ± 1
Myrtenol	27.54	1193	1194	0.83 ± 0.04
cis-Verbenone	28.12	1205	1204	3.0 ± 0.2
trans-Carveol	28.6	1215	1215	1.3 ± 0.1
Citronellol	29.03	1224	1223	0.48 ± 0.01
cis-Carveol	29.15	1227	1226	0.3 ± 0.2
Carvone	29.76	1240	1239	0.43 ± 0.03
Piperitone	30.25	1250	1249	0.27 ± 0.02
Phellandral	31.22	1271	1273	0.15 ± 0.02
p-Cymen-7-ol	31.95	1286	1289	0.45 ± 0.01
β-Elemene	36.53	1388	1389	0.10 ± 0.01
β-Caryophyllene	37.75	1416	1417	0.139 ± 0.003
cis-Thujopsene	38.25	1428	1429	0.346 ± 0.005
β-cadiene	42.01	1521	1520	0.138 ± 0.002
Oplopanone	49.97	1745	1739	0.21 ± 0.01
Total identified				95.14%
Monoterpene hydrocarbons				2.78%
Oxygen-containing monoterpenes				89.1%
Sesquiterpene hydrocarbons				0.9%
Others				1.54%
Not identified				5.64%

^a^ LRI: linear retention index determined on a DB-5 MS fused silica column relative to a series of n-alkanes (C8–C40). ^b^ LRI: linear retention index reported in the literature [31]. ^c^ Relative % is given as average ± standard deviation.

**Table 3:** Antibacterial activity of *J. communis* essential oil obtained by steam distillation (clinical bacteria). MIC and MBC values (% *v*/*v*).

	Controls							
	*J. communis*		Ampicillin		Imipenem		Vancomycin	
			20 mg/mL		1 mg/mL		1 mg/mL	
Clinical Bacteria	MIC	MBC	MIC	MBC	MIC	MBC	MIC	MBC
**Gram-negative bacteria**								
*Escherichia coli*	>2.5	>2.5	<0.15	<0.15	<0.0078	<0.0078	n.t	n.t.
*Klebsiella pneumoniae*	>2.5	>2.5	10	20	<0.0078	<0.0078	n.t	n.t.
*Morganella morganii*	>2.5	>2.5	20	>20	<0.0078	<0.0078	n.t	n.t.
*Proteus mirabilis*	>2.5	>2.5	<0.15	<0.15	<0.0078	<0.0078	n.t	n.t.
*Pseudomonas aeruginosa*	>2.5	>2.5	>20	>20	0.5	1	n.t.	n.t.
**Gram-positive bacteria**								
*Enterococcus faecalis*	>2.5	>2.5	<0.15	<0.15	n.t.	n.t.	<0.0078	<0.0078
*Listeria monocytogenes*	>2.5	>2.5	<0.15	<0.15	<0.0078	<0.0078	n.t	n.t.
MRSA	>2.5	>2.5	<0.15	<0.15	n.t.	n.t.	0.25	0.25

MIC: minimum inhibitory concentration; MBC: minimum bactericidal concentration; MRSA: methicillin-resistant *Staphylococcus aureus*; n.t.: not tested.

**Table 4:** Antibacterial activity of *J. communis* essential oil obtained by steam distillation (foodborne bacteria).

	Controls							
	*J. communis*		Streptomycin		Methicillin		Ampicillin	
	(% *v*/*v*)		1 mg/mL		1 mg/mL		20 mg/mL	
Food Bacteria	MIC	MBC	MIC	MBC	MIC	MBC	MIC	MBC
**Gram-negative bacteria**								
*Enterobacter cloacae*	>2.5	>2.5	0.007	0.007	n.t.	n.t	0.15	0.15
*Escherichia coli*	>2.5	>2.5	0.01	0.01	n.t.	n.t.	0.15	0.15
*Pseudomonas aeruginosa*	>2.5	>2.5	0.06	0.06	n.t.	n.t.	0.63	0.63
*Salmonella enterica*	2.5	>2.5	0.007	0.007	n.t.	n.t.	0.15	0.15
*Yersinia enterocolitica*	>2.5	>2.5	0.007	0.007	n.t.	n.t.	0.15	0.15
**Gram-positive bacteria**								
*Bacillus cereus*	2.5	>2.5	0.007	0.007	n.t.	n.t.	n.t.	n.t.
*Listeria monocytogenes*	>2.5	>2.5	0.007	0.007	n.t.	n.t.	0.15	0.15
*Staphylococcus aureus*	>2.5	>2.5	0.007	0.007	0.007	0.007	0.15	0.15

MIC: minimum inhibitory concentration; MBC: minimum bactericidal concentration; n.t. not tested.

**Table 5:** Antifungal activity of *J. communis* essential oil obtained by steam distillation.

	*J. communis*		Ketoconazole	
	(% *v*/*v*)		1 mg/mL	
Fungi	MIC	MFC	MIC	MFC
*Aspergillus brasiliensis*	2.5	>2.5	0.06	0.125
*Aspergillus fumigatus*	2.5	>2.5	0.5	1

MIC: minimum inhibitory concentration; MFC: minimum fungicidal concentration.

**Table 6:** Biochar compliance with EU regulation n.2019/1009 for fertilising products, as CMC 14.

Parameter	CMC14 Threshold	*J. communis* Biochar
PAH16 (mg/kg d.b.)	<6.0	1.36
PCDD/F (ng _WHO toxicity equivalents_/kg d.b.)	<20.0	<1.0
PCB non-dioxin-like (mg/kg d.b.)	<0.8	0.001
Cl^−^ (g/kg d.b.)	<30.0	0.02
Tl (mg/kg d.b.) *	<2.0	<1.0
H/C_ORG_ molar ratio **	<0.7	0.356

d.b.: Dry basis; * Reg. (EU) 2019/1009: with pyrolysis additives > 5% of loaded fresh mass; ** Reg. (EU) 2019/1009: on dry and ash-free fraction for material with a C_ORG_ content < 50%.

**Table 7:** Biochar compliance with EU regulation n.2019/1009 for fertilising products, as PFC 3(A).

Parameter	PFC 3(A) Threshold	*J. communis* Biochar
Organic carbon (% *w*/*w* d.b.)	>7.5	87.3
Dry matter (% *w*/*w* d.b.)	>20.0	98.2
Cadmium (mg/kg d.b.)	<2.0	0.4
Hexavalent chromium (mg/kg d.b.)	<2.0	<0.2
Mercury (mg/kg d.b.)	<1.0	<0.1
Nickel (mg/kg d.b.)	<50.0	b.d.l.
Lead (mg/kg d.b.)	<120.0	b.d.l.
Inorganic arsenic (mg/kg d.b.)	<40.0	<0.2 (as Total As)
Copper (mg/kg d.b.)	<300.0	57.0
Zinc (mg/kg d.b.)	<800.0	9.0
*Salmonella* spp. (CFU/25 g)	absent	n.a.
*Escherichia coli* or Enterococcaceae (CFU/g)	<1000	n.a.

% *w*/*w*: % Weight/weight; d.b.: dry basis; CFU: colony-forming units; b.d.l.: below detection limit; n.a.: not available.

**Table 8:** Physical–chemical characterisation of *J. communis* pellets as cat litter and reference values.

Parameter	Absorbing Plant-Based Cat Litter Feasibility Reference Values	*J. communis* Pellets
Appearance	Clear colours usually related to hygiene sensation	Spotted dark brown cylindrical particles
Dry particle size distribution (mm)	1.0–8.0	5.6–6.3
Moisture content (% *w*/*w*, w.b.)	Max. 14.0	10.5
Dust content (a.u.)	As low as possible	7
Bulk density (g/L) (w.m.)	450–650	465
Water absorption (% *w*/*w*, w.b.)	>180	267
Durability (%*w*/*w*, w.b.)	>95.0	88.9
Ammonia absorption (NH_3_ ppm) (4 h)	As low as possible	0

% *w*/*w*: % Weight/weight; w.b.: wet basis; a.u.: arbitrary units; w.m: wet matter.

## Data Availability

Not applicable.

## Sample Availability

Samples of the compounds are not available from the authors.

## Abbreviations

a.u.	arbitrary units
a.s.l.	above sea level
b.d.l.	below detection limit
CAA	cellular antioxidant activity
CFU	colony forming units
CMC	component material category
CORG	organic carbon
d.b.	dry basis
GI50	extract concentration that provides 50% of cell growth inhibition
IPCC	Intergovernmental Panel on Climate Change
LRI	linear retention index
MBC	minimum bactericidal concentration
MFC	minimum fungicidal concentration
MIC	minimum inhibitory concentration
MRSA	Methicillin-resistant *Staphylococcus aureus*
n.a.	not available
n.t.	not tested
PAH	polycyclic aromatic hydrocarbon
PCDD/F	polychlorinated-p-dioxins and furans
PFC	product function category
RP	reducing power
*v*/*v*	volume/volume
w.b.	wet basis
w.m	wet matter
*w*/*w*	weight/weight

## Appendix A

**Table A1:** Characterisation of *J. communis* biochar—proximate and ultimate analyses.

Parameter	Result	Unit of Measure
Moisture	1.80	% *w*/*w*, a.r.
Ashes	8.65	% *w*/*w*, d.b.
Volatiles	12.98	% *w*/*w*, d.b.
Fixed Carbon	78.37	% *w*/*w*, d.b.
C	87.76	% *w*/*w*, d.b.
H	2.61	% *w*/*w*, d.b.
N	0.74	% *w*/*w*, d.b.
Cl	0.02	% *w*/*w*, d.b.
S	0.03	% *w*/*w*, d.b.
Inorganic C	0.49	% *w*/*w*, d.b.
pH	7.5	
Conductivity	395.1	µS/cm

% *w*/*w*: % Weight/weight; a.r.: as received; d.b.: dry basis.

**Table A2:** Characterisation of *J.communis* biochar—Elements content.

Parameter	Result	Unit of Measure
Al	265	mg/kg d.b.
B	b.d.l.	mg/kg, d.b.
Ba	88	mg/kg, d.b.
Ca	15,455	mg/kg, d.b.
Co	b.d.l.	mg/kg, d.b.
Cr	b.d.l.	mg/kg, d.b.
Cu	57	mg/kg, d.b.
Fe	241	mg/kg, d.b.
K	7035	mg/kg, d.b.
Li	b.d.l.	mg/kg, d.b.
Mg	1666	mg/kg, d.b.
Mn	422	mg/kg, d.b.
Mo	b.d.l.	mg/kg, d.b.
Na	1543	mg/kg, d.b.
Ni	b.d.l.	mg/kg, d.b.
P	b.d.l.	mg/kg, d.b.
Pb	b.d.l.	mg/kg, d.b.
Si	514	mg/kg, d.b.
Ti	26	mg/kg, d.b.
V	b.d.l.	mg/kg, d.b.
Zn	9	mg/kg, d.b.
Cr VI	<0.2	mg/kg, d.b.
Cd	0.40	mg/kg, d.b.
As	<2	mg/kg, d.b.
Hg	<0.1	mg/kg, d.b.
Tl	<1	mg/kg, d.b.

d.b.: Dry basis.

**Table A3:** Characterisation of *J. communis* biochar—Organic pollutants.

Parameter	Result	Unit of Measure
**PAH**		
NAPHTALENE	<0.05	mg/kg, d.b.
ACENAPHTHYLENE	<0.05	mg/kg, d.b.
ACENAFTENE	<0.05	mg/kg, d.b.
FLUORENE	<0.05	mg/kg, d.b.
PHENANTHRENE	0.43	mg/kg, d.b.
ANTHRACENE	0.10	mg/kg, d.b.
FLUORANTHENE	0.21	mg/kg, d.b.
PYRENE	0.24	mg/kg, d.b.
BENZO(A)ANTHRACENE	0.08	mg/kg, d.b.
CRISENE	0.07	mg/kg, d.b.
BENZO(B + J + K)FLUORANTHENE	0.05	mg/kg, d.b.
BENZO(E)PYRENE	<0.05	mg/kg, d.b.
BENZO(A)PYRENE	0.05	mg/kg, d.b.
PERILENE	<0.05	mg/kg, d.b.
INDENO(1,2,3,CD)PYRENE	<0.05	mg/kg, d.b.
DIBENZO(AH)ANTHRACENE	<0.05	mg/kg, d.b.
BENZO(GHI)PERILENE	<0.05	mg/kg, d.b.
DIBENZO(A,L)PYRENE	<0.05	mg/kg, d.b.
DIBENZO(A,I)PYRENE	<0.05	mg/kg, d.b.
DIBENZO(A,E)PYRENE	<0.05	mg/kg, d.b.
DIBENZO(A,H)PYRENE	<0.05	mg/kg, d.b.
SUM PAH	1.36	mg/kg, d.b.
**Polychlorinated Dibenzodioxins and Polychlorinated Dibenzofurans**		
2,3,7,8 TETRA CDD	<1	ng/kg, d.b.
1,2,3,7,8 PENTA CDD	<5	ng/kg, d.b.
1,2,3,4,7,8 ESA CDD	<5	ng/kg, d.b.
1,2,3,6,7,8 ESA CDD	<5	ng/kg, d.b.
1,2,3,7,8,9 ESA CDD	<5	ng/kg, d.b.
1,2,3,4,6,7,8 EPTA CDD	<5	ng/kg, d.b.
OCTA CDD	<10	ng/kg, d.b.
2,3,7,8 TETRA CDF	<1	ng/kg, d.b.
1,2,3,7,8 PENTA CDF	<5	ng/kg, d.b.
2,3,4,7,8, PENTA CDF	<5	ng/kg, d.b.
1,2,3,4,7,8 ESA CDF	<5	ng/kg, d.b.
1,2,3,6,7,8 ESA CDF	<5	ng/kg, d.b.
2,3,4,6,7,8 ESA CDF	<5	ng/kg, d.b.
1,2,3,7,8,9 ESA CDF	<5	ng/kg, d.b.
1,2,3,4,6,7,8 EPTA CDF	<5	ng/kg, d.b.
1,2,3,4,7,8,9 EPTA CDF	<5	ng/kg, d.b.
OCTA CDF	<10	ng/kg, d.b.
Equivalent Toxicity according to WHO-TEQ	<1	ng WHO-TEQ/kg d.b.
**Polychlorinated Biphenyls**		
T3CB-28	0.00027	mg/kg, d.b.
T4CB-52	0.00023	mg/kg, d.b.
P5CB-95	0.00008	mg/kg, d.b.
P5CB-101	0.00010	mg/kg, d.b.
P5CB-99	0.00003	mg/kg, d.b.
P5CB-110	0.00008	mg/kg, d.b.
P5CB-118	0.00006	mg/kg, d.b.
H6CB-151	<0.0001	mg/kg, d.b.
H6CB-149	0.00003	mg/kg, d.b.
H6CB-146	<0.0001	mg/kg, d.b.
H6CB-153	0.00006	mg/kg, d.b.
H6CB-138	0.00004	mg/kg, d.b.
H6CB-128	<0.0001	mg/kg, d.b.
H7CB-187	<0.0001	mg/kg, d.b.
H7CB-183	<0.0001	mg/kg, d.b.
H7CB-177	<0.0001	mg/kg, d.b.
H7CB-180	<0.0001	mg/kg, d.b.
H7CB-170	<0.0001	mg/kg, d.b.
SUM PCB	0.00101	mg/kg, d.b.
**WHO-PCB (dioxin-like)**		
T4CB-77	<0.0001	ng/kg, d.b.
T4CB-81	<0.0001	ng/kg, d.b.
P5CB-105	0.00	ng/kg, d.b.
P5CB-114	<0.0001	ng/kg, d.b.
P5CB-118	<0.0001	ng/kg, d.b.
P5CB-123	<0.0001	ng/kg, d.b.
P5CB-126	<0.0001	ng/kg, d.b.
H6CB-156	<0.0001	ng/kg, d.b.
H6CB-157	<0.0001	ng/kg, d.b.
H6CB-167	<0.0001	ng/kg, d.b.
H6CB-169	<0.0001	ng/kg, d.b.
H7CB-189	<0.0001	ng/kg, d.b.
Equivalent Toxicity according to WHO-TEQ	<0.01	ng WHO-TEQ/kg, d.b.

d.b.: Dry basis.

**Table A4:** Characterisation of *J. communis* biochar—Porosity and water sorption capacity.

Parameter	Result	Unit of Measure
BET and DFT analysis results for micro-mesopore range		
Specific surface area (BET)	61	m^2/^g
Specific pore volume (DFT)	0.04	cm^3^/g
Mercury porosimeter analysis results for meso-macropore range		
Specific surface area	40	m^2^/g
Specific intrusion volume	1.59	cm^3^/g
Porosity	66.5	%
Water-holding capacity results		
Water specific retention	1.42	ml_H2O/_g_CHAR_
